# Expansion of the split hygromycin toolkit for transgene insertion in *Caenorhabditis elegans*

**DOI:** 10.17912/micropub.biology.001091

**Published:** 2024-01-29

**Authors:** Megan J Moerdyk-Schauwecker, Erin K Jahahn, Zachariah I Muñoz, Kristin J Robinson, Patrick C Phillips

**Affiliations:** 1 Institute of Ecology and Evolution, University of Oregon, Eugene, Oregon, United States

## Abstract

Engineered sites for genetic transformation have simplified transgene insertion in *Caenorhabditis elegans* . These strategies include our split hygromycin system ​(Stevenson et al. 2020)​ which allows for integration-specific selection of transgenes. Here we have expanded the split hygromycin selection system to include two additional chromosomal locations, both of which are permissive for germline expression, as well as engineered landing pads in three additional natural isolates. Corresponding guide and empty repair template plasmids are also available for each of these sites.

**Figure 1. The expanded split hygromycin toolkit:**
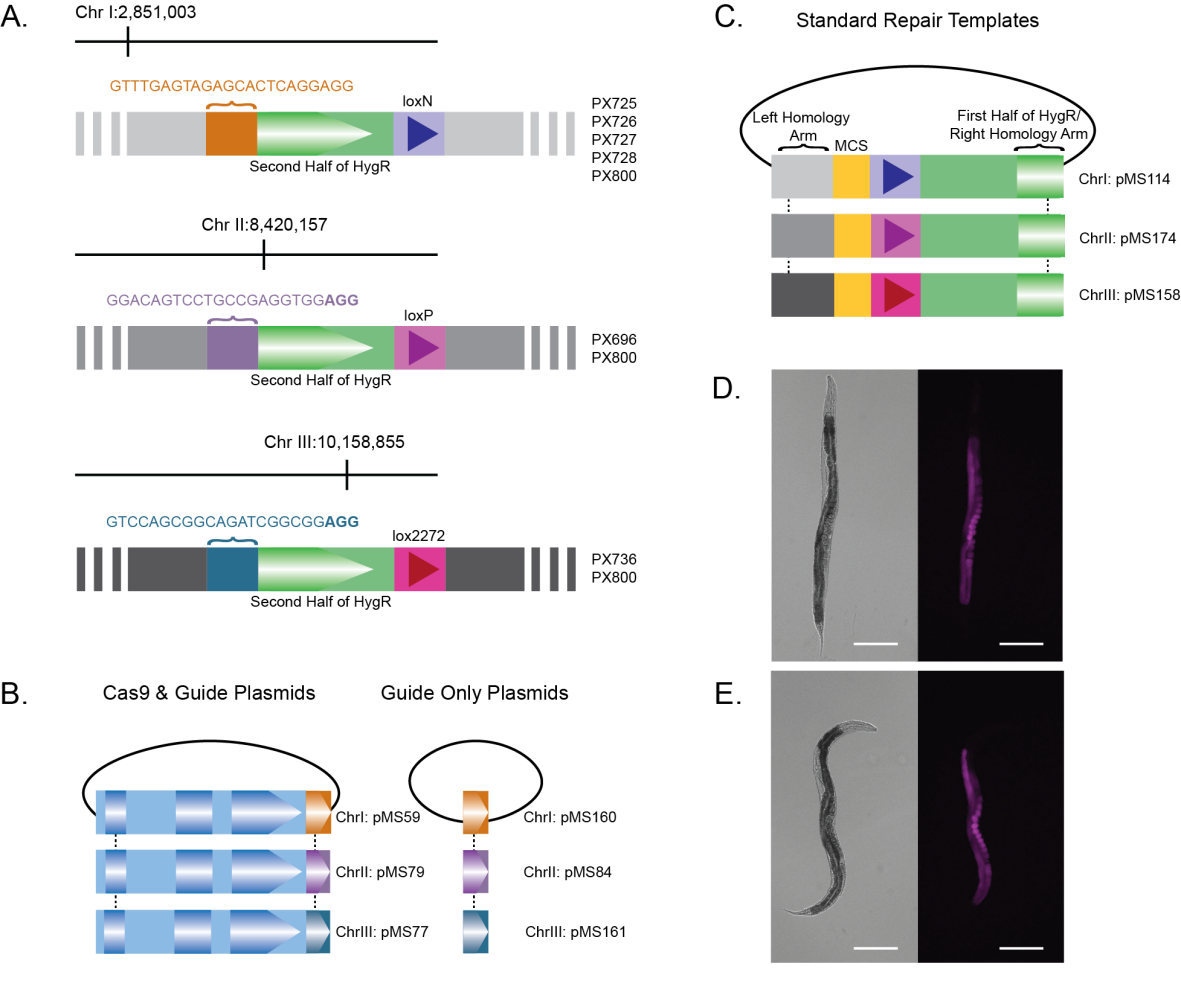
(A) Landing pads containing a synthetic guide site, the 3’ portion of the hygromycin resistance gene and a lox site were integrated into three different locations on three different chromosomes at the locations indicated. Each synthetic guide site is targeted by (B) its own Cas9 plus guide plasmid or guide only plasmid. (C) Empty repair template plasmids with multiple cloning sites for cargo insertion were also made to correspond to each landing pad. Integrated *mex-5p::mKate2* reporters were used to demonstrate germline permissiveness of the (D) chromosome I and (E) chromosome III landing pad sites. Scale bar = 200µm.

## Description

Several strategies have been developed utilizing engineered “safe harbor” or “landing pad” sites for transgene insertion in *Caenorhabditis elegans* ​(Silva-García et al. 2019; Nonet 2020, 2021, 2023; Stevenson et al. 2020, 2023; Yang et al. 2020; Mouridi et al. 2022; Malaiwong et al. 2023)​. These sites facilitate efficient introduction of transgenes by providing a means for selection of integrants and/or simplifying the required insertion template. We have previously demonstrated split hygromycin selection as a simple, rapid and highly generalizable system for transgene insertion in *C. elegans* ​ (Stevenson et al. 2020) ​. This system splits the hygromycin resistance ( *HygR* ) gene between the landing pad site and the repair template, meaning that only individuals with an integrated transgene, and not array bearing individuals, have resistance to the drug hygromycin B and can therefore be identified by a simple live/dead screen. Here we expand this system (1) to additional chromosomal locations, allowing for generation of more complex transgenics; and (2) to several commonly used *C. elegans* natural isolates, allowing for generation and comparison of transgenics in multiple genetic backgrounds.

Previously we introduced a single copy, split hygromycin landing pad into chromosome II of N2 ( PX696 ) ​(Stevenson et al *.* 2020)​. Here, using the same self-excising cassette (SEC) based CRISPR strategy, we also introduced a single copy, split hygromycin landing pad into chromosome I of N2 at the same intergenic region occupied by the *ttTi4348* transposon utilized in MosSCI ​(Frøkjær-Jensen et al. 2012)​ and into chromosome III of N2 between the *nac-1* and *K08E5.5* genes, thereby creating strains PX725 and PX736 respectively ( Figure 1A ). Both of these locations have also been previously utilized for direct transgene insertion ​ (Kasimatis et al. 2018, 2022) ​. Each of these three landing pads contain the 3’ portion of the coding sequence for the *HygR* gene and a *unc-54* 3’ UTR flanked by a lox site. This allows for repair template integration specific selection of transgenic individuals and for subsequent optional removal of *HygR* by expression of Cre recombinase ​ (Stevenson et al. 2020) ​. A unique lox sequence is used for each landing pad site, allowing them to be used together without triggering interchromosomal recombination ​ (Lee and Saito 1998; Langer et al. 2002) ​.

Each landing pad also contains a unique, high-efficiency synthetic guide site ​ (Stevenson et al. 2020) ​, that is targeted for insertion of transgenic cargo. This guide site can be targeted by injection of either a plasmid expressing the guide plus Cas9 or a plasmid expressing guide alone plus an independent source of Cas9 ( Figure 1B ). This Cas9 can come from either a separate plasmid, purified protein or a strain expressing integrated Cas9 [for example ​ (Zhang et al. 2018; Schwartz et al. 2021) ​]. We demonstrated that the landing pads can be used in conjunction with integrated Cas9 by crossing PX696 and PX725 with the Cas9 bearing strain EG9882 to create PX783 and PX784 respectively. PX736 is incompatible with EG9882 due to both strains possessing a lox2272 site. A *rpl-28p::mKate2* transcriptional reporter was then integrated into the PX783 and PX784 strains by CRISPR to generate PX798 and PX792 . In screening for integrants, we noticed a high frequency of removal of the *HygR* gene used for selection, even in the absence of heat shock, likely due to leaky expression of the *hsp-16.41p::Cre* construct also present in EG9882 . As such, it may be possible to achieve higher integration efficiencies if integrated Cas9 strains lacking Cre are used.

For each landing pad a corresponding empty repair template plasmid was also made ( Figure 1C ). These plasmids each contain homology to the landing pad on either side of the cut site, a multiple cloning site (MCS) to facilitate linearization of the vector (although this can also be done by PCR), the *rps-0* promoter proceeded by a lox sequence matching that in the landing pad, and the 5’ portion of the *HygR* coding sequence. While we created generic, empty repair templates, repair templates could also be customized for specific purposes. In principle, it should be possible to combine the split hygromycin selection with SEC ​ (Dickinson et al. 2015) ​, thereby bypassing the need for an outside source of Cre for removal of the *HygR* gene. While it does indeed work to include the additional SEC components in the repair template, for reasons that are unclear, transgene insertion using this repair template is not only significantly lower than the base split hygromycin system, but also lower than that achieved using the original SEC protocol.

Since all three landing pads have been designed to be compatible with each other and are individually targetable, they can be combined to make more complex recombinants either through crosses or by sequentially inserting genes into a strain containing two or more landing pads, although in this case removal of the restored *HygR* gene is required to restore the drug-sensitive background prior to the next round of editing. In principle, it is also possible to take a co-CRISPR like approach and simultaneously target two or more sites as editing at one site increases the probability of editing at additional sites even when using large repair templates ​ (Stevenson et al. 2023) ​. To facilitate these latter approaches, we have generated PX729 , which contains the chromosome I and II sites, and PX800 , which contains all three landing pad sites, in an N2 background.

One of the advantages of landing pads generated via CRISPR is that as long as the guide site sequence is conserved in the genome, the same reagents can be used to integrate a landing pad into any *C. elegans* genetic background. This creates a path for a more general study of background specific effects in individual alleles [i.e. epistasis ​ (Phillips 2008) ​]. Similarly, since the landing pad creates a standardized guide and homology arm set, transgenes can also be integrated into any particular landing pad using the same reagents regardless of genetic background. To demonstrate this and to create landing pads in several commonly used strains as a resource for the community, we integrated the chromosome I landing pad into MY16 , CB4856 and JU775 to create PX726 , PX727 and PX728 respectively. These strains have been used in multiple quantitative trait locus (QTL) mapping experiments ​ (Andersen and Rockman 2022) ​, as well as being core component of the *Caenorhabditis* Intervention Testing Program (CITP) ​ (Lucanic et al. 2017) ​.

Finally, one limitation to transgenesis in *C. elegans* is that transgene expression can be restricted in the germline, and this effect is based in part on chromosome location ​(Frøkjær-Jensen et al. 2012)​. Therefore, it is important to determine the germline permissiveness of any new transgene insertion site. The chromosome II landing pad has been previously shown to be permissive for germline expression ​ (Stevenson et al. 2020) ​. To test the germline permissiveness of chromosome I and III landing pads, a *mex-5p::mKate2* reporter was integrated into each. In both cases, robust fluorescence could be detected in the germline ( Figure 1D and E ), indicating that these landing pads are germline permissive. For the chromosome I landing pad, this is consistent with previous work using the *ttTi4348* MosSCI site ​(Frøkjær-Jensen et al. 2012)​.

Overall, these new strains and reagents expand the *C. elegans* split hygromycin toolkit and allow integration of genetic elements at multiple independent sites in the genome and across multiple genetic backgrounds.

## Methods

*Worm maintenance*

All strains were maintained at 15⁰C on nematode growth media (NGM) seeded with *Escherichia coli* OP50 unless otherwise specified.

*Plasmids*

Guide plus Cas9 plasmids have been previously described ​(Kasimatis et al *.* 2018, 2022; Stevenson et al *.* 2020)​. Guide only plasmids were generated by site directed mutagenesis of pZCS11 ​ (Stevenson et al. 2023) ​ using the Q5 site-directed mutagenesis kit [New England Biolabs (NEB), E0554S)]. Empty repair templates were generated from an insert-containing repair template using site directed mutagenesis to remove the insert and replace it with an MCS.

The landing pad insertion plasmids and insert-containing repair templates were generated by Gibson cloning using NEBuilder HiFi DNA assembly master mix (NEB, E2621). Cloning fragments were made either by restriction digest of existing vectors or by PCR using Q5 high-fidelity 2x master mix (NEB, M0492).

For the chromosome I landing pad insertion plasmid (pMS87), the backbone and homology arms were amplified from pMS30 ​ (Kasimatis et al. 2018) ​ and the split HygR landing pad and SEC with loxN sites added by PCR were amplified from pMS70 ​ (Stevenson et al. 2020) ​. For the chromosome III landing pad insertion plasmid (pMS110), the backbone and homology arms were amplified from pMS63 ​ (Kasimatis et al. 2022) ​ and the split HygR landing pad and SEC with lox2272 sites added by PCR were amplified from pMS72 ​(Stevenson et al *.* 2020)​.

The chromosome II repair template containing *rpl-28p::mKate2* (pMS81) has been previously described ​ (Stevenson et al. 2020) ​. pMS81 was also used as the source of the plasmid backbone, split *HygR* and *rpl-28p::mKate2* source for the corresponding chromosome I repair template (pMS143.1), while the genomic homology arm was amplified from pMS87 and the loxN site was added by synthesis.

For both the chromosome I and chromosome III *mex-5p::mKate2* repair templates (pMS144 and pMS145 respectively), *mex-5p* was amplified from genomic DNA, *mKate2* was amplified from pMS81 and the *tbb-2 3’ UTR* was amplified from pCFJ421 ​(Frøkjær-Jensen et al. 2012)​. The remaining components of pMS144 were amplified from pMS114. For pMS145 the genomic homology arm was amplified from pMS63 while with backbone and split HygR site were amplified from pMS81 with addition of the lox2272 site by PCR.

All plasmids were purified using the ZR plasmid miniprep classic kit (Zymo Research, D4016) and verified by sequencing prior to use. Plasmid sequences are available through Addgene or upon request.

*Transgenic generation*

To make single copy insertions of the landing pads, young adult hermaphrodites of the desired background strain were injected with a mixture consisting of 10ng/µl repair template plasmid (pMS87 or pMS110), 50ng/µl guide plus Cas9 plasmid (pMS18 or pMS62) and 2.5ng/µl pCFJ421 that served as an array marker. Following injection, worms were maintained at 25°C. Selection, screening, and SEC removal were done as in ​ (Dickinson et al. 2015) ​.

For insertion of germline reporters into the landing pads, young adult PX725 or PX736 hermaphrodite were injected with a mixture consisting of 15ng/µl repair template plasmid (pMS144 or pMS145) and 50ng/µl guide plus Cas9 plasmid (pMS59 or pMS77). To test landing pad insertions using integrated Cas9, young adult PX783 or PX784 hermaphrodites were injected with a mixture consisting of 15ng/µl repair template plasmid (pMS81 or pMS143.1) and 25ng/µl guide only plasmid (pMS84 or pMS160)

In both cases, following injection, worms were maintained at 25°C and dosed with hygromycin B at a final concentration of 250µg/ml approximately 48 hours later. Five to seven days after dosing, candidate worms were singled and genotyped by PCR.

Genotyping of candidate individuals was done by single worm PCR using OneTaq quick-load 2x master mix with standard buffer (NEB, M0486). All transgene insertions were confirmed by Sanger sequencing. Sequences of genotyping/sequencing primers are available upon request. All strains are available through CGC, CaeNDR or upon request.

*Microscopy*

Microscopy images were obtained with the 10x objective on an Olympus IX inverted fluorescent microscope using the Olympus cellSens Dimension 2.3 capture software and an Andor Zyla sCMOS camera. Worms were mounted on a 2% agarose pad and immobilized using 100mM sodium azide. Fluorescent images were captured using the mCherry settings with an exposure time of 10 ms. Light images were captured using the DIC filter set. Fluorescent images were recolored using ImageJ2 version 2.9.0.

## Reagents

**Table:** 

**Strain**	**Strain Background**	**Genotype**	**Reference/Generated**	**Availability**
N2 - PD1073	NA	Wild Isolate	​​ (Yoshimura et al. 2019) ​	Upon request
MY16	NA	Wild Isolate	​​ (Crombie et al. 2023) ​	CaeNDR
CB4856	NA	Wild Isolate	​​ (Crombie et al. 2023) ​	CaeNDR
JU775	NA	Wild Isolate	​​ (Crombie et al. 2023) ​	CaeNDR
EG9882	N2	*oxTi1127* [ *mex-5* p::Cas9(+ *smu-2* introns):: *tbb-2* 3'UTR + *hsp-16.41* p::Cre:: *tbb-2* 3'UTR + *myo-2* p::2xNLS::cyOFP:: *let-858* 3'UTR + lox2272] III	​​ (Schwartz et al. 2021) ​	CGC
PX696	N2	*fxIs10* [synthetic guides site5::∆HygR:: *unc-54* 3' UTR::LoxP, II:8420157]	​​ (Stevenson et al. 2020) ​	CGC
PX725	N2	*fxSi8* [synthetic guide site1::3'∆HygR:: *unc-54* 3' UTR::LoxN, I:2851003]	This work	CGC
PX726	MY16	*fxSi9* [synthetic guide site1::3'∆HygR:: *unc-54* 3' UTR::LoxN, I:2851003]	This work	CGC
PX727	CB4856	*fxSi10* [synthetic guide site1::3'∆HygR:: *unc-54* 3' UTR::LoxN, I:2851003]	This work	CGC
PX728	JU775	*fxSi11* [synthetic guide site1::3'∆HygR:: *unc-54* 3' UTR::LoxN, I:2851003]	This work	CGC
PX736	N2	*fxSi13* [synthetic guide site3::3'∆HygR:: *unc-54* 3' UTR::Lox2272, III:10158855]	This work	CGC
PX729	N2	*fxIs10* [synthetic guide site5::∆HygR:: *unc-54* 3' UTR::LoxP, II:8420157]; *fxSi8* [synthetic guide site1::3'∆HygR:: *unc-54* 3' UTR::LoxN, I:2851003]	This work	Upon request
PX800	N2	*fxIs10* [synthetic guide site5::∆HygR:: *unc-54* 3' UTR::LoxP, II:8420157]; *fxSi8* [synthetic guide site1::3'∆HygR:: *unc-54* 3' UTR::LoxN, I:2851003]; *fxSi13* [synthetic guide site3::3'∆HygR:: *unc-54* 3' UTR::Lox2272, III:10158855]	This work	Upon request
PX783	N2	*fxIs10* [synthetic guide site5::∆HygR:: *unc-54* 3' UTR::LoxP, II:8420157]; *oxTi1127* [ *mex-5* p::Cas9(+ *smu-2* introns):: *tbb-2* 3'UTR + *hsp-16.41* p::Cre:: *tbb-2* 3'UTR + *myo-2* p::2xNLS::cyOFP:: *let-858* 3'UTR + lox2272] III	This work	Upon request
PX784	N2	*fxSi8* [synthetic guide site1::3'∆HygR:: *unc-54* 3' UTR::LoxN, I:2851003]; *oxTi1127* [ *mex-5* p::Cas9(+ *smu-2* introns):: *tbb-2* 3'UTR + *hsp-16.41* p::Cre:: *tbb-2* 3'UTR + *myo-2* p::2xNLS::cyOFP:: *let-858* 3'UTR + lox2272] III	This work	Upon request
PX793	N2	*fxSi43* [ *mex-5* p::mKate2:: *tbb-2* 3'UTR + loxN+ *rps-0* p:HygR:: *unc-54* 3' UTR + loxN, I:2851003]	This work	Upon request
PX797	N2	*fxSi45* [ *mex-5* p::mKate2:: *tbb-2* 3'UTR + lo2272+ *rps-0* p:HygR:: *unc-54* 3' UTR + lox2272, III:10158855]	This work	Upon request
PX792	N2	*fxSi42* [ *rpl-28* p::mKate2:: *unc-54* 3'UTR + loxN+ *rps-0* p:HygR:: *unc-54* 3' UTR + loxN, I:2851003]; *oxTi1127* [ *mex-5* p::Cas9(+ *smu-2* introns):: *tbb-2* 3'UTR + *hsp-16.41* p::Cre:: *tbb-2* 3'UTR + *myo-2* p::2xNLS::cyOFP:: *let-858* 3'UTR + lox2272] III	This work	Upon request
PX798	N2	*fxSi46* [ *rpl-28* p::mKate2:: *unc-54* 3'UTR + loxP+ *rps-0* p:HygR:: *unc-54* 3' UTR + loxP, II:8420157]; *oxTi1127* [ *mex-5* p::Cas9(+ *smu-2* introns):: *tbb-2* 3'UTR + *hsp-16.41* p::Cre:: *tbb-2* 3'UTR + *myo-2* p::2xNLS::cyOFP:: *let-858* 3'UTR + lox2272] III	This work	Upon request

**Table:** 

**Plasmid**	**Purpose**	**Insert**	**Generated**	**Availability**
pMS87	Landing pad insertion plasmid for ChrI	5'HA + synthetic guide site1 + 3'ΔHygR:: *unc-54* 3' UTR + loxN + SEC + loxN + 3'HA	This work	Addgene
pMS110	Landing pad insertion plasmid for ChrIII	5'HA + synthetic guide site3 + 3'ΔHygR: *unc-54* 3' UTR + lox2272 + SEC + lox2272 + 3'HA	This work	Addgene
pMS18	Cas9 + guide plasmid for inserting ChrI landing pad	*eef-1A.1* p::Cas9 + U6p::GAAATCGCCGACTTGCGAGG	​​ (Kasimatis et al. 2018) ​	Addgene
pMS62	Cas9 + guide plasmid for inserting ChrIII landing pad	*eef-1A.1* p::Cas9 + U6p::GTCGTTCTTCCGTTCTCGGG	​​ (Kasimatis et al. 2022) ​	Addgene
pMS114	Empty repair plasmid for ChrI	5'HA + MCS + loxN + *rps-0* p::5'ΔHygR	This work	Addgene
pMS178	Empty repair plasmid for ChrII	5'HA + MCS + loxP + *rps-0* p::5'ΔHygR	This work	Addgene
pMS158	Empty repair plasmid for ChrIII	5'HA + MCS + lox2272 + *rps-0* p::5'ΔHygR	This work	Addgene
pMS59	Cas9 + guide plasmid targeting ChrI landing pad	*eef-1A.1* p::Cas9 + U6p::GTTTGAGTAGAGCACTCAGG	​​ (Stevenson et al. 2020) ​	Addgene
pMS79	Cas9 + guide plasmid targeting ChrII landing pad	*eef-1A.1* p::Cas9 + U6p::GGACAGTCCTGCCGAGGTGG	​​ (Stevenson et al. 2020) ​	Addgene
pMS77	Cas9 + guide plasmid targeting ChrIII landing pad	*eef-1A.1* p::Cas9 + U6p::GTCCAGCGGCAGATCGGCGG	​​ (Stevenson et al. 2020) ​	Addgene
pMS160	Guide only plasmid targeting chrI landing pad	U6p::GTTTGAGTAGAGCACTCAGG	This work	Addgene
pMS84	Guide only plasmid targeting chrII landing pad	U6p::GGACAGTCCTGCCGAGGTGG	​​ (Stevenson et al. 2023) ​	Addgene
pMS161	Guide only plasmid targeting chrIII landing pad	U6p::GTCCAGCGGCAGATCGGCGG	This work	Addgene
pMS143.1	Reporter for insertion into chrI landing pad	5'HA + *rpl-28* p::mKate2:: *unc-54* 3'UTR + *rps-0* p::5'ΔHygR	This work	Upon request
pMS81	Reporter for insertion into chrII landing pad	5'HA + *rpl-28* p::mKate2:: *unc-54* 3'UTR + loxP + *rps-0* p::5'ΔHygR	​​ (Stevenson et al. 2020) ​	Addgene
pMS144	Germline reporter for insertion into chrI landing pad	5'HA + *mex-5* p::mKate2:: *tbb-2* 3'UTR + loxN + *rps-0* p::5'ΔHygR	This work	Upon request
pMS145	Germline reporter for insertion into chrIII landing pad	5'HA + *mex-5* p::mKate2:: *tbb-2* 3'UTR + lox2272 + *rps-0* p::5'ΔHygR	This work	Upon request
pCFJ421	Array marker; source for cloning components	*myo-2* p::GFP::H2B	​​(Frøkjær-Jensen et al. 2012)​	Addgene
pMS30	Source for cloning components	5'HA + *pie-1* p::AtTIR::mRuby:: *unc-54* 3'UTR +loxP + SEC + loxP +3'HA	​​ (Kasimatis et al. 2018) ​	Upon request
pMS63	Source for cloning components	5'HA + *hsp16.41* p:: *peel-1* :: *tbb-2* 3'UTR + *rpl-28* p::mKate2:: *unc-54* 3'UTR + *rps-0* p::HygR:: *unc-54* 3'UTR + 3' HA	​​ (Kasimatis et al. 2022) ​	Upon request
pMS70	Source for cloning components	5'HA + synthetic guide site1 + 3'ΔHygR:: *unc-54* 3' UTR + loxP + SEC + loxP + 3'HA	​​ (Stevenson et al. 2020) ​	Upon request
pMS72	Source for cloning components	5'HA + synthetic guide site3 + 3'ΔHygR:: *unc-54* 3' UTR + loxP + SEC + loxP + 3'HA	​​ (Stevenson et al. 2020) ​	Upon request
pZCS11	Empty vector for guide expression	U6p::Scaffold	​​ (Stevenson et al. 2023) ​	Upon request
